# Public health education: the status of health and health-related physical activity courses in Texas community colleges

**DOI:** 10.3389/fpubh.2023.1199734

**Published:** 2023-08-24

**Authors:** Garry G. Ladd

**Affiliations:** Department of Health and Human Performance, Parker University, Dallas, TX, United States

**Keywords:** public health education, health, wellness, physical fitness, college

## Abstract

**Background:**

There is an inverse relationship between disease and both health literacy and physical literacy. Courses taken during the completion of degrees at community colleges help to prepare students to be productive members of society by teaching knowledge, skills, and abilities needed for employment and good citizenship. Coursework in public health education and health-related behaviors should be included in the overall community college curriculum. The purpose of this study was to determine the status of Health/Wellness/Physical Fitness Lecture (HWPFL) and Health-Related Physical Activity (HRPA) courses in Texas community colleges.

**Methods:**

A review of the institutional websites of Texas community colleges (N = 50) was performed to access information regarding HWPFL and HRPA courses and graduation requirements. Information regarding the Texas Core Curriculum was reviewed for any local additions to the state mandated requirements that students are required to complete prior to the attainment of Associate in Arts (AA) and Associate in Science (AS) degrees.

**Results:**

Individual colleges were grouped into those with <5,000 students (*N* = 21), those having 5,000–10,000 students (*N* = 16), and those with >10,000 students (*N* = 13). Three (6%) institutions require a HWPFL course for AA and AS degrees and 49 (98%) had such courses available for elective credit. Two (4%) colleges require an HRPA course for AA and AS degrees whereas 47 (94%) offer such courses for elective credit. There was one college that only offers a HWPFL course. One college did not offer either HWPFL or HRPA courses. There were many identical courses offered by various colleges for required and elective credit.

**Conclusion:**

Requiring health, wellness, and health-related physical activity requirements in Texas community colleges can be a policy solution for improving health and physical literacy in students. This research study demonstrated the prevalence of health, wellness, and health-related physical activity requirements in the 50 public community colleges in the state of Texas. Offering courses in health, wellness, and health-related physical activity as required or elective courses for graduation from Texas community colleges is a means through which to develop health literacy and physical literacy in students resulting in a positive influence on public health.

## 1. Introduction

Community colleges can have a positive effect on public health. During the transition from high school to college or into the workforce, young adults move from being under the influence and guidance of their parents or guardians regarding their health decisions and health related behaviors to becoming more independent in their choices that impact health both positively and negatively (1). This transition also affects legal and financial responsibilities for health and healthcare. As these young adults become independent and attend college or enter the workforce, they become more autonomous regarding their health-related decisions and behaviors. Those attending community colleges during this transition are preparing for vital roles in society by seeking advanced education and skills. The colleges help to prepare the students by having a set of degree requirements to be followed to ensure that knowledge and skills are learned to prepare for adulthood. The purpose of this study was to determine the status of Health/Wellness/Physical Fitness Lecture (HWPFL) and Health-Related Physical Activity (HRPA) courses in Texas community colleges by examining the specific graduation requirements of each institution that can influence individual and public health.

Two areas that affect individual and community public health are health literacy and physical literacy, both of which can be enhanced during attendance at community colleges. Health literacy is defined as the degree to which individuals have the ability to find, understand, and use information and services to inform health-related decisions and actions for themselves and others (2). Being that community college students are transitioning to a time in life when they will undertake increased responsibility for their own health, it makes sense to help them prepare by offering courses within the curriculum to help with that transition. The same can be true for physical literacy, which is defined as a multidimensional concept that describes a holistic foundation for physical activity engagement (3).

Texas public community colleges serve a vital role in the economy of the state by helping to develop the workforce and prepare students for further academic pursuits. Community colleges were specifically created to expand access to higher education to the public (4). In the state of Texas, the 50 public community college districts fulfill an important role by meeting the specific educational and vocational needs of their service areas. Community colleges are one of the best venues through which adults can receive education and experiences in promoting their own health and that of their families, communities, and workplace. Community colleges can help adults learn and acquire the knowledge, skills, and abilities to become and stay healthy and productive throughout their lifetime. The knowledge, skills, and abilities needed to increase the healthy years of life and to reduce sickness and disability can be taught, learned, and practiced at community colleges and well into adult life and by doing so, improve personal and public health outcomes.

The Texas Higher Education Coordinating Board (THECB) requires that public community colleges follow a state mandated Texas Core Curriculum (5). The THECB requires that Associate of Arts (AA) and Associate in Science (AS) degrees include a curriculum of general education classes of 42 credit hours of courses designed to provide a breadth of knowledge to Texas community college graduates. Associate of Applied Science (AAS) degrees require a minimum of 15 h of coursework to provide this breadth of knowledge. To that end, all degrees require at least one course in humanities/fine arts, social/behavioral sciences, and natural science/mathematics. The THECB also mandates a component in communication, history, and government for AA and AS degrees designed to transfer to a four-year college or university. The public community colleges may as an option, add institutional graduation requirements in addition to students meeting the Texas Core Curriculum requirements (5).

When college and university students engage in academic courses, campus recreational activities, and lifestyles that enhance health, wellness, and health-related physical activity, the benefits gained during the college years can continue well into adulthood. Previous studies have shown that some of the benefits from participating in a college physical activity course include improved academic performance and retention (6, 7), decreased signs of psychological symptoms of anxiety, depression, and stress (8–10), and increased social connection with others (11). When physical activity courses are required to meet graduation requirements, research has shown that this can positively affect the knowledge and attitudes of alumni and positively affect their lifelong behaviors (12). In addition, when college and university students are required to specifically take a lifetime physical fitness course, there is a positive effect on the health behaviors of students (13).

The leading causes of death in the state of Texas include heart disease as the number one cause of death and stroke is number three (14), both of these are cardiovascular diseases that can be prevented in part by increasing levels of physical activity. The financial burden for hospitalization for heart disease and stroke in 2016 for the state of Texas was 23 billion dollars and is expected to rise (15). Heart disease and stroke are largely preventable. It is a priority in the state of Texas to establish and promote environments that support the reduction of heart disease and stroke through positive lifestyle behaviors including healthy eating, physical activity, preventing and controlling diabetes, breastfeeding, and increasing tobacco-free lifestyles (14). Each of these health-related behaviors can be learned through taking appropriate HWPFL and HRPA courses at community colleges in Texas.

The use of campus recreational facilities and taking elective physical activity courses has been shown to attract students who are already motivated and engaged in physical activity (14). Many college students are intimidated by campus fitness and recreational facilities and programs and as a result, remain physically inactive (16, 17). These physically inactive students may benefit the most from institutional academic requirements that include completing health, wellness, and health-related physical activity courses. Requiring health, wellness, and health-related physical activity courses ensures that quality instruction, evidence-based health-related physical activity, and scheduled time for such activities can enhance health and physical literacy and help motivate these students to continue lifetime health related behaviors (18).

The purpose of this study was to determine the status of HWPFL and HRPA courses in Texas community colleges by examining the specific required and elective AA and AS degree graduation requirements of each institution. Knowing the current state of HWPFL and HRPA courses in Texas community colleges can help drive policy making decisions that can impact health and well-being in current and future students. Increasing health and physical literacy in community college students could have a life-long impact on quality and quantity of life of individuals, families, and communities and also help to address gaps, issues, and controversies that can influence individual and public health.

## 2. Materials and methods

### 2.1. Participants

A complete list of Texas Community Colleges was obtained through the Texas Higher Education Coordinating Board website (19). Inclusion criteria was community colleges that are public and offered Associate in Arts (AA) and Associate in Science (AS) degree programs. The total sample for this study consisted of 50 Texas public community colleges. Institutional websites were accessed for each institution and information regarding the Texas Core Curriculum was reviewed for any local additions to the state mandated requirements that students were required to complete prior to attainment of the AA or AS degree. There were no exclusion criteria used for this study.

### 2.2. Procedure

This cross-sectional descriptive study utilized protocols found elsewhere including searching institutional websites and academic catalogs to determine if HWPFL and/or HWPFL courses were offered, required for graduation and/or offered as elective credit, and the course names and descriptions (20–22). Publicly available information regarding graduation requirements and course offerings was reviewed for each institution. The Institutional Review Board deemed the study to be exempt due to the use of publicly available data and that no human contact or resource was used in the collection of data. Each community college’s website and most recent official college catalog were searched for information regarding the respective AA and AS transfer degrees and graduation requirements. Information sought included: (1) the name of the institution, (2) the inclusion of the Texas Core Curriculum, (3) any specific additional requirements by the individual colleges, (4) if HWPFL, and/or HRPA courses were required for graduation or offered as elective credits, (5) if the courses were primarily didactic or activity based, and (6) the course names that were categorized as HWPFL or HRPA courses. The primary key words used for searches at institutional websites included health, wellness, physical education, kinesiology, physical activity, graduation requirements, and elective credit. The review of institutions was completed from January 2023 to March 2023. Limitations to this study was that there was a sole investigator and that enrollment data for specific courses at individual institutions was not available for inclusion.

Health/Wellness/Physical Fitness Lecture courses were identified as those that included a minimum of one contact hour of lecture. Some of the HWPFL courses do include physical activity in addition to lecture. First Aid courses were included due to the inclusion of public health topics that promote safety and preventative educational methods that reduce the risk of injury, accidents, and lifestyle related diseases. These include the avoidance of hazards within the environment, rendering intelligent assistance in case of accident or sudden illness, and to develop skills necessary for the immediate and temporary care of the victim. Drug education courses were included due to public health concerns and consequences over illicit and prescription drug use, misuse, and abuse.

Health-related physical fitness has been defined as those components of fitness that can contribute to overall health and well-being and include cardiorespiratory endurance, body composition, muscular strength, muscular endurance, and flexibility (23). Health-Related Physical Activity courses are those that are primarily activity based with no lecture and only activity contact hours as garnered from the course description. For HRPA courses, course descriptions were used to verify the activity and the attainment or improvement of a minimum of one component of health-related fitness versus instruction, practice, and sport-based courses. Activity courses that are described as instructional, practice, or sport-based were not used for this study with the reason being that many of these types do not contribute to nor identify any of the health-related components of fitness in the course description. Swimming courses, Yoga, Pilates, Tai Chi, Kickboxing, and other activity courses were included only if the course description included terms such as conditioning, fitness, or a specific health-related fitness component such as cardiorespiratory, strength, or flexibility. Courses for varsity athletic team participation or strength/conditioning for athletics were not included in this study. When courses offered more than one level of progression, they were only counted once. For example, when a college offered courses such as beginning weight training, intermediate weight training, or advanced weight training, those courses were only included as weight training and only counted once.

### 2.3. Analysis

Data collected were summarized using descriptive statistics including institutional enrollments, means, ranges, and percentages of institutions offering HWPFL and/or HRPA courses for graduation and/or elective credit. The main focus of the data analysis was to determine if health, wellness, or health-related physical activity courses were offered as a mandated graduation requirement for AA and AS transfer degrees, were offered as elective credit which could be applied towards graduation requirements, or not offered at all. Responses were recorded and tabulated into Microsoft Excel (Microsoft 365, Microsoft Corporation, Redmond, WA). Descriptive statistics were performed using Microsoft Excel. Data analysis was conducted by determining the current number of Texas public community colleges based on student population. After reviewing the institutional websites and college catalogs, the total number of programs that required these courses and offered elective credit courses was also calculated. The percentage was then calculated by reporting the total number required out of the total number of institutions that met the inclusion criteria (*N* = 50).

## 3. Results

For data analysis, individual colleges were grouped into those with <5,000 students (*N* = 21; Table 1), 5,000 to 10,000 students (*N* = 16; Table 2), and those >10,000 students (*N* = 13; Table 3). The total student population reported for public community colleges in Texas during fall of 2021 was 616,537 (mean = 12,331). Student populations were based on the Fall 2021 semester student enrollment as reported by THECB Institutional Resumes online database (19).

**Table 1:** Texas community colleges population < 5,000 (*N* = 21).

Community college	Population Fall 2021
Clarendon College	1,401
Western Texas College	1,430
Frank Phillips College	1,583
Galveston College	1,870
Ranger College	2,262
Vernon College	2,358
Panola College	2,475
Northeast Texas Community College	2,823
Cisco College	3,090
Victoria College	3,125
Texarkana College	3,573
Hill College	3,826
Grayson College	3,865
Brazosport College	3,914
Coastal Bend College	3,927
Howard County Junior College	3,930
Temple College	3,959
College of the Mainland Community College	4,133
Angelina College	4,249
Paris Junior College	4,297
Alvin Community College	4,970

Source: Texas Higher Education Coordinating Board. Institutional Resumes (2022) (https://apps.highered.texas.gov/resumes/).

**Table 2:** Texas community colleges population 5,000–1,000 (*N* = 16).

Community college	Population Fall 2021
Midland College	5,045
Kilgore College	5,081
Trinity Valley Community College	5,426
Wharton County Junior College	5,479
Southwest Texas Junior College	5,594
Weatherford College	5,645
Navarro College	6,456
Central Texas College	6,641
Lee College	7,159
McLennan Community College	7,329
Texas Southmost College	7,527
North Central Texas College	7,574
Odessa College	7,943
South Plains College	8,925
Amarillo College	9,140
Laredo Community College	9,968

Source: Texas Higher Education Coordinating Board. Institutional Resumes (2022) (https://apps.highered.texas.gov/resumes/).

**Table 3:** Texas community colleges population > 10,000 (*N* = 13).

Community college	Population fall 2021
Del Mar College	10,395
Tyler Junior College	11,898
Blinn College	17,077
El Paso Community College	23,824
South Texas College	28,184
San Jacinto Community College	31,577
Collin County Community College	32,603
Austin Community College	32,890
Tarrant County College	36,264
Houston Community College	38,861
Alamo Community College	50,201
Dallas County Community College	61,925
Lone Star College System	62,846

Source: Texas Higher Education Coordinating Board. Institutional Resumes (2022) (https://apps.highered.texas.gov/resumes/).

Overall, regarding HWPFL courses for Texas Community College, three (6%) colleges require a HWPFL course to meet AA or AS degree graduation requirements and 49 (98%) offer a HWPFL course that can be taken as an elective. One does not offer a required or elective HWPFL course that can be used to complete degree requirements.

For the colleges with a student population of <5,000, there are none that require a HWPFL course for graduation and all but one have a minimum of one HWPFL course available as an elective. For colleges with a student population of 5,000–10,000, one requires a HWPFL course for graduation and all but one have a minimum of one HWPFL course available as an elective. For colleges with a student population of >10,000, two require a HWPFL course for graduation and all offer a minimum of one HWPFL course available as an elective (Tables 4–6).

**Table 4:** Texas community colleges population < 5,000 (*N* = 21) health/wellness/physical fitness courses required or elective.

Community college (listed in order of population)	Required health/wellness/physical fitness lecture (yes/no)	Elective health/wellness/physical fitness lecture (yes/no)
Clarendon College	No	Yes
Western Texas College	No	Yes
Frank Phillips College	No	Yes
Galveston College	No	Yes
Ranger College	No	Yes
Vernon College	No	Yes
Panola College	No	Yes
Northeast Texas Community College	No	Yes
Cisco College	No	Yes
Victoria College	No	Yes
Texarkana College	No	Yes
Hill College	No	Yes
Grayson College	No	Yes
Brazosport College	No	No
Coastal Bend College	No	Yes
Howard County Junior College	No	Yes
Temple College	No	Yes
College of the Mainland Community College	No	Yes
Angelina College	No	Yes
Paris Junior College	No	Yes
Alvin Community College	No	Yes

**Table 5:** Texas community colleges population 5,000–1,000 (*N* = 16) health/wellness/physical fitness courses required or elective.

Community college (listed in order of population)	Required health/wellness/physical fitness lecture course (yes/no)	Elective health/wellness/physical fitness lecture course (yes/no)
Midland College	No	Yes
Kilgore College	No	Yes
Trinity Valley Community College	No	Yes
Wharton County Junior College	No	Yes
Southwest Texas Junior College	No	Yes
Weatherford College	Yes	Yes
Navarro College	No	Yes
Central Texas College	No	Yes
Lee College	No	Yes
McLennan Community College	No	Yes
Texas Southmost College	No	Yes
North Central Texas College	No	Yes
Odessa College	No	Yes
South Plains College	No	Yes
Amarillo College	No	No
Laredo Community College	No	Yes

**Table 6:** Texas community colleges population > 10,000 (*N* = 13) health/wellness/physical fitness courses required or elective.

Community college (listed in order of population)	Required health/wellness/physical fitness lecture (yes/no)	Elective health/wellness/physical fitness lecture (yes/no)
Del Mar College	No	Yes
Tyler Junior College	No	Yes
Blinn College	No	Yes
El Paso Community College	No	Yes
South Texas College	No	Yes
San Jacinto Community College	No	Yes
Collin County Community College	No	Yes
Austin Community College	No	Yes
Tarrant County College	Yes	Yes
Houston Community College	No	Yes
Alamo Community College	No	Yes
Dallas County Community College	No	Yes
Lone Star College System	Yes	Yes

Regarding HRPA courses for Texas Community Colleges, two (4%) colleges require a HRPA course that can be met by taking a two or three credit hour kinesiology class to meet AA or AS degree graduation requirements. Forty-seven (94%) offer a HRPA course that can be taken as an elective. One does not offer a required or an elective HRPA course that can be used to complete degree requirements.

For the colleges with a student population of <5,000, there is one that requires a HRPA course for graduation and all but three have a minimum of one HRPA course available as an elective. For colleges with a student population of 5,000–10,000, one requires an HRPA course for graduation, and all have a minimum of one HRPA course available as an elective. For colleges with a student population of >10,000, none require a HRPA course for graduation, and all offer a minimum of one HRPA course as an elective (Tables 7–9).

**Table 7:** Texas community colleges population < 5,000 (*N* = 21) health-related physical activity courses required or elective.

Community college population <5,000 (listed in order of population)	Required health-related physical activity course (yes/no)	Elective health-related physical activity course (yes/no)
Clarendon College	No	Yes
Western Texas College	No	Yes
Frank Phillips College	No	No
Galveston College	No	Yes
Ranger College	No	Yes
Vernon College	No	Yes
Panola College	Yes (3 h kinesiology activity)	Yes
Northeast Texas Community College	No	Yes
Cisco College	No	Yes
Victoria College	No	Yes
Texarkana College	No	Yes
Hill College	No	Yes
Grayson College	No	Yes
Brazosport College	No	No
Coastal Bend College	No	No
Howard County Junior College	No	Yes
Temple College	No	Yes
College of the Mainland Community College	No	Yes
Angelina College	No	Yes
Paris Junior College	No	Yes
Alvin Community College	No	Yes

**Table 8:** Texas community colleges population 5,000–1,000 (*N* = 16) health-related physical activity courses required or elective.

Community college population 5,000–1,000 (listed in order of population)	Required health-related physical activity course (yes/no)	Elective health-related physical activity course (yes/no)
Midland College	No	Yes
Kilgore College	No	Yes
Trinity Valley Community College	No	Yes
Wharton County Junior College	No	Yes
Southwest Texas Junior College	No	Yes
Weatherford College	No	Yes
Navarro College	No	Yes
Central Texas College	No	Yes
Lee College	No	Yes
McLennan Community College	No	Yes
Texas Southmost College	No	Yes
North Central Texas College	No	Yes
Odessa College	No	Yes
South Plains College	Yes (2 h kinesiology activity)	Yes
Amarillo College	No	Yes
Laredo Community College	No	Yes

**Table 9:** Texas community colleges population > 10,000 (*N* = 13) health-related physical activity courses required or elective.

Community college population > 10,000 (listed in order of population)	Required health-related physical activity course (yes/no)	Elective health-related physical activity course (yes/no)
Del Mar College	No	Yes
Tyler Junior College	No	Yes
Blinn College	No	Yes
El Paso Community College	No	Yes
South Texas College	No	Yes
San Jacinto Community College	No	Yes
Collin County Community College	No	Yes
Austin Community College	No	Yes
Tarrant County College	No	Yes
Houston Community College	No	Yes
Alamo Community College	No	Yes
Dallas County Community College	No	Yes
Lone Star College System	No	Yes

Many of the institutions share similar courses as required or elective HWPFL or HRPA courses. The four most common HWPFL courses are First Aid (*N* = 42), Personal and Community Health (*N* = 40), Concepts of Physical Fitness (*N* = 36), and Drug Use and Abuse (*N* = 31). The four most common HRPA courses are Weight Training (*N* = 38), Yoga (*N* = 29), Conditioning (*N* = 27), and Walking (*N* = 22). The names of the HWPFL offered at each institution are included in Tables 10–12. The names of the HRPA courses offered at each institution are included in Tables 13–15.

**Table 10:** Texas community colleges population < 5,000 (*N* = 21) health/wellness/physical fitness lecture course names.

Community college population < 5,000 (listed in order of population)	Health/wellness/physical fitness course names
Clarendon College	Personal/Community Health, First Aid, Drug Use and Abuse
Western Texas College	Personal/Community Health, First Aid, Drug Use and Abuse
Frank Phillips College	Personal/Community Health, Concepts of Physical Fitness
Galveston College	First Aid, Personal/Community Health, Concepts of Fitness, Drug Use and Abuse
Ranger College	First Aid, Personal/Community Health, Concepts of Fitness, Drug Use and Abuse
Vernon College	Personal/Community Health, Introduction to Physical Fitness and Wellness, First Aid
Panola College	Introduction to Physical Fitness and Wellness, Personal/Community Health, First Aid
Northeast Texas Community College	Wellness for a Lifetime, Health Education, First Aid, Concepts of Physical Fitness
Cisco College	First Aid and Safety Education, Drug Use and Abuse, First Aid, Personal and Community
Victoria College	Concepts of Physical Fitness
Texarkana College	Personal/Community Health
Hill College	Personal/Community Health, First Aid, Concepts of Physical Fitness, Drug Use and Abuse
Grayson College	Personal and Community Health, First Aid, Concepts of Physical Fitness, Drug Use and Abuse
Brazosport College	None
Coastal Bend College	Personal/Community Health, First Aid, Concepts of Physical Fitness, Drug Use and Abuse
Howard County Junior College	Personal/Community Health, First Aid, Concepts of Physical Fitness, Drug Use and Abuse
Temple College	Personal /Community Health, First Aid, Concepts of Physical Fitness
College of the Mainland Community College	Weight Control, Personal/Community Health, First Aid
Angelina College	Personal and Community Health, First Aid, Concepts of Physical Activity, Drug Use and Abuse
Paris Junior College	Personal/Community Health, First Aid, Concepts of Physical Fitness, Drug Use and Abuse
Alvin Community College	Alvin Community College

**Table 11:** Texas community colleges population 5,000–1,000 (*N* = 16) health/wellness/physical fitness lecture course names.

Community college population 5,000–1,000 (listed in order of population)	Health/wellness/physical fitness lecture course names
Midland College	Introduction to Physical Fitness and Wellness, First Aid
Kilgore College	Personal/Community Health, Safety-First Aid, Concepts of Physical Fitness, Drug Use and Abuse
Trinity Valley Community College	Introduction to Physical Fitness and Sport, Personal/Community Health, First Aid, Concepts of Physical Fitness
Wharton County Junior College	Introduction to Physical Fitness and Wellness, Personal/Community Health, First Aid, Drug Use and Abuse
Southwest Texas Junior College	Personal and Community Health, First Aid, Concepts of Physical Fitness
Weatherford College	Introduction to Physical Fitness and Wellness, Concepts of Physical Fitness, Personal and Community Health, First Aid
Navarro College	Introduction to Physical Fitness and Wellness, Personal/Community Health, First Aid, Concepts of Physical Fitness, Drug Use and Abuse
Central Texas College	Personal and Community Health, First Aid, Concepts of Physical Fitness
Lee College	Personal/Community Health, Concepts of Physical Fitness, Drug Use and Abuse
McLennan Community College	First Aid, Concepts of Physical Fitness, Wellness and Lifestyle
Texas Southmost College	First Aid, Concepts of Physical Fitness, Intro to Physical Fitness and Wellness
North Central Texas College	Personal/Community Health, First Aid, Concepts of Physical Fitness, Drug Use and Abuse
Odessa College	Introduction to Physical Fitness and Sport, First Aid, Personal/Community Health, Concepts of Physical Fitness, Drug Use and Abuse
South Plains College	Fitness and Wellness, First Aid and Safety, Drug Use and Abuse
Amarillo College	None
Laredo Community College	Personal/Community Health, First Aid, Concepts of Physical Fitness, Drug Use and Abuse

**Table 12:** Texas community colleges population > 10,000 (*N* = 13) health/wellness/physical fitness lecture course names.

Community college population > 10,000 (listed in order of population)	Health/wellness/physical fitness lecture course names
Del Mar College	Personal/Community Health I: Health and Lifestyle, First Aid, Concepts of Physical Fitness, Drug Use and Abuse
Tyler Junior College	Concepts of Physical Fitness, Personal/Community Health, First Aid, Drug Use and Abuse, Introduction to Physical Fitness and Wellness
Blinn College	Personal/Community Health, First Aid, Concepts of Physical Fitness, Drug Use and Abuse
El Paso Community College	Wellness and Health Promotion, First Aid, Concepts of Physical Fitness
South Texas College	Introduction to Physical Fitness and Wellness, Personal/Community Health, First Aid, Concepts of Physical Fitness, Drug Use and Abuse
San Jacinto Community College	Introduction to Physical Fitness and Wellness, Exercise for Health and Fitness, Introduction to Physical Fitness and Wellness, Personal/Community Health, First Aid, Concepts of Physical Fitness, Drug Use and Abuse
Collin County Community College	Personal/Community Health, First Aid, Concepts of Physical Fitness
Austin Community College	Personal Health, First Aid, Concepts of Physical Fitness, Drug Use and Abuse
Tarrant County College	Introduction to Physical Fitness and Wellness, Personal and Community Health, First Aid, Concepts of Physical Fitness, Drug Use and Abuse
Houston Community College	Personal/Community Health, Concepts of Physical Fitness, First Aid, Drug Use and Abuse
Alamo Community College	Drug Use and Abuse, Personal/Community Health, First Aid
Dallas County Community College	Personal/Community Health
Lone Star College System	Personal/Community Health, First Aid, Drug Use and Abuse

**Table 13:** Texas community colleges population < 5,000 (*N* = 21) health-related physical activity course names.

Community college population < 5,000 (listed in order of population)	Health-related physical activity course names
Clarendon College	Weight Lifting, Walking for Fitness, Physical Fitness, Cardiovascular Fitness, Introduction to Physical Fitness and Sport
Western Texas College	P.E. Activity for Freshmen (weight training, yoga, body conditioning)
Frank Phillips College	None
Galveston College	Walking and Jogging, Yoga, Strength and Conditioning, Stretch and Tone, Weight Training, Introduction to Physical Fitness and Sport
Ranger College	Activity in Physical Education, Weight Training
Vernon College	Weight Training and Conditioning, Physical Conditioning, Fitness Walking, Aquatic Conditioning, Spinning
Panola College	Weight Training, Body Conditioning, Zumba Fitness, Fitness Through Walking, Running and Jogging, Physical Education Boot Camp, Kickboxing, Bungee Fitness, Barre Above, Yoga/Pilates, Aerial Yoga, Pilates, Spin
Northeast Texas Community College	Weight Training and Aerobic Activity, Weight Training, Body Conditioning, Walking/Jogging, Spinning, Fitness Boxing
Cisco College	Physical Training, Yoga, Walking, Yoga, Physical Training, Walking Physical Training, Weights, Physical Training
Victoria College	Weight Training, Yoga, Group Physical Fitness/Weight Training and Conditioning
Texarkana College	Weight Training, Cross Training for Fitness and Weight Control, Rhythmic Aerobics, Yoga/Pilates for Fitness, Yin Yoga, Bench Stepping, Cardio Kickboxing, Body Sculpting
Hill College	Aerobics, Weight Lifting, Walking/Jogging, Swimming and Conditioning, Zumba, Body Stretching and Sculpting, Tai Chi, Intro to Wellness/Fitness
Grayson College	Slimnastics, Weight Training and Conditioning, Jogging and Conditioning, Yoga, Introduction to Physical Fitness and Wellness
Brazosport College	None
Coastal Bend College	None
Howard County Junior College	Weight Training, Aerobics, Running, Aqua Aerobics, Lifetime Fitness, Yoga
Temple College	Jogging/Walking/Fitness, Weight Training, Physical Conditioning
College of the Mainland Community College	Weight Training, Hatha Yoga, Aerobic Run/Walk, Aerobic Cross Training, Aerobic Kickboxing, Introduction to Physical Fitness and Wellness
Angelina College	Exercise and Conditioning, Weight Training and Conditioning, Aerobics, Introduction to Strength and Cardiovascular Fitness, Low-Impact Muscle Conditioning, Advanced Weight Training, High Intensity Interval Training, Introduction to Physical Fitness and Wellness
Paris Junior College	Aerobics, Introduction to Wellness (activity class), Swim Conditioning, Maintenance of Wellness (activity class), Aqua Aerobics, Weight Training
Alvin Community College	Physical Fitness and Weight Training, Individual and Dual Sports – Fitness and Wellness

**Table 14:** Texas community colleges population 5,000–1,000 (*N* = 16) health-related physical activity course names.

Community college population 5,000-10,000 (listed in order of population)	Health-related physical activity course names
Midland College	Individualized Fitness, Pilates, Yoga
Kilgore College	Weight Training, Exercise and Fitness, Walking and Jogging, Yoga
Trinity Valley Community College	Conditioning and Weight Training, Aerobics and Physical Fitness
Wharton County Junior College	Aerobics, Weight Lifting/Circuit Training
Southwest Texas Junior College	Dance for Conditioning, Conditioning, Jogging for Fitness, Weight Training
Weatherford College	Weight Training and Conditioning, Pilates, Yoga, Jogging, Zumba
Navarro College	Aerobics, Fitness Dance, Jogging, Weightlifting, Cardiokickboxing, Pilates, Fitness Walking, Hatha Yoga
Central Texas College	Aerobics, Fitness Walking, Spin Bike, Physical Conditioning, Water Fitness, Weight Training, Yoga, Jogging, Pilates, Zoomba Cardio, Bootcamp Fitness, Eagle Fit, Barbell Fitness
Lee College	Exercise, Pilates, Aerobic Components, Water Aerobics, Weight Training, Yoga, Walking/Jogging, Introduction to Physical Fitness and Wellness
McLennan Community College	Introduction to Wellness and Health, Introduction to Physical Fitness and Wellness, Figure and Weight Control, Aerobic Dance and Exercise, Weight Training, Aerobic Weight Training, Walking for Life, Stretching and Flexibility, Contemporary Health, Pilates and Yoga Exercise
Texas Southmost College	Intro to Physical Fitness and Wellness
North Central Texas College	Physical Conditioning, Jogging/Walking, Weight Training/Jogging
Odessa College	Lifestyle Assessment and Modification, Jogging/Walking, Physical Conditioning Aerobic, Weight Training, Cardio Kickboxing, Zumba, Spinning, Yoga
South Plains College	Water Aerobics, Deep Water Aerobics, Indoor Cycling, Yoga, Walk Run Jog, Cardio Core Conditioning, Weight Training for Women, Weight Training for Men, Cardio Kickboxing, Co-Ed Weight Lifting, Aerobics, Conditioning
Amarillo College	Lifetime Fitness
Laredo Community College	Pilates, Weight Training, Hatha Yoga, Water Aerobics, Indoor Cycling, Aerobics, Jogging, Conditioning Body Sculpting, Power Walking, Introduction to Physical Fitness and Wellness

**Table 15:** Texas community colleges population > 10,000 (*N* = 13) health-related physical activity course names.

Community college population > 10,000 (listed in order of population)	Health-related physical activity course names
Del Mar College	Introduction to Physical Fitness and Wellness
Tyler Junior College	Physical Conditioning, Weight Training
Blinn College	Ab/Glute Conditioning, Aerobics, Fitness Conditioning, Pilates, Aerobic Running, Weight Training, Yoga, Aerobic Walking, strength Training for Athletic Performance
El Paso Community College	Conditioning, Jogging, Weight Training, Introduction to Physical Fitness and Wellness
South Texas College	Fitness Walking, Fitness and Motor Development, Weight Training and Conditioning, Yoga and Flexibility Training, Aerobic Dance
San Jacinto Community College	Jogging, Water Aerobics, Aerobic Activities, Advanced Aerobics, Weight Training, Yoga, Fitness Swimming, Fitness Walking, Kickboxing for Fitness
Collin County Community College	Weight Training, Walking and Fitness, Hatha Yoga, Water Aerobics, Swimming Conditioning, Aerobic Dance, Jogging and Fitness, High Intensity Interval Training, Introduction to Physical Fitness and Wellness
Austin Community College	Cardio Conditioning, Aqua Fitness, Conditioning-Stretching/Flexibility, Conditioning-Walk/Jog, Weight Training, Yoga, Aerobics
Tarrant County College	Aerobic Fitness, Conditioning, Swim Conditioning, Walk or Jog, Water Aerobics, Water Exercise, Weight Training, Yoga, Pilates, Kickboxing
Houston Community College	Yoga, Jogging, Physical Fitness Training, Weight Training and Conditioning, Introduction to Physical Fitness and Wellness
Alamo Community College	Aerobics, Cardio Kickboxing, Latin Cardio Dance, Core Training, Jogging, Walking, Indoor Cycling, Weight Training, High Intensity Interval training, Cardio Boot Camp, Pliyo/Barre Fitness, Pilates, Yoga, Conditioning
Dallas County Community College	Conditioning Exercise, Weight Training, Jogging, Walking for Fitness, Aerobics, Aquatic Fitness, Cycling, Triathlon Fitness, Lifetime Fitness and Wellness, Zumba Fitness, Yoga, Pilates, Cardiovascular Fitness
Lone Star College System	Introduction to Physical Fitness and Wellness (required), Weight Training, Jogging, Aerobics, Aqua Aerobics, Yoga, Tai Chi

## 4. Discussion

According to the Texas Education Code, health and physical education must be offered in grades K-12 as part of the required curriculum in Texas schools (24, 25). As stated in the TEC, a local school district may provide instruction through a variety of settings, add elements at its discretion, but must not delete or omit instruction in the enrichment curriculum which does include physical education. The gap between requiring health and physical education in grades K-12 in Texas and requiring health, wellness, and health-related physical activity in Texas community colleges is wide moving from 100% of K-12 Schools requiring physical education to only 6% of community colleges requiring a HWPFL course and 4% requiring HRPA course to meet graduation requirements. Although 100% of Texas high school students must meet a mandated physical education requirement for graduation, specifically that requirement is attaining 1.0 credits of physical education while in high school while the maximum physical education credits is capped at 4.0 (26).

Texas community colleges are well equipped to help meet national health goals as found in the Healthy People project, and more specifically, the goals and objectives of Healthy People 2030. The Healthy People Project in the United States provides public health objectives with defined 10-year benchmarks (27). Healthy People 2030 (HP2030) identifies public health priorities to help individuals, organizations, and communities in the United States to improve health and well-being and builds upon the first four decades of the program (28). The HP2030 initiative is the fifth iteration. There are six HP2030 objectives identified as being related to health literacy (29, 30).

When comparing Texas community colleges to two and four-year institutions that require physical education for graduation, a much lower number of Texas community colleges require physical education and physical activity courses that can be found nationwide. Previous studies have shown a decline in requiring physical education from an all-time high of 97% in the 1920s and 1930s to an all-time low of 39.55% in 2010 (20). The number of Texas community colleges that require a physical education or physical activity course for graduation ranges from only 6% requiring a HWPFL course to 4% requiring a HRPA course. When comparing Texas community colleges to colleges and universities in Colorado, the difference is not as much as that seen nationally with only 15.6% of institutions in Colorado requiring physical education for graduation (21). Texas community colleges also lag behind community colleges in Oregon, where all community colleges require at least a partial physical education requirement (18). The percentage of Texas community colleges that require a HWPFL or HRPA course for graduation is much lower than what is seen nationally in post-secondary institutions which includes that 56.2% do not mandate physical education courses but 31.7% do fully and 12% partially require undergraduate students to complete a physical education course for graduation (22).

Although most Texas community colleges do not have a graduation requirement for HWPFL courses, the current status of HWPFL courses in Texas community colleges shows there is strong evidence of offering various courses that can enhance the health and wellbeing of students and their families now and in the future. Forty-nine (98%) of the colleges offer a minimum of one HWPFL course with most offering more than one. Although only three (6%) require such a course to meet AA or AS degree graduation requirements, almost all offer HWPFL courses that can be taken as elective credit. Community colleges in Texas are in a very good position to educate students either through required or elective courses to improve and/or reinforce health literacy in students for their immediate as well as long-term needs.

Texas community colleges are also in a good position to help meet national health goals for meeting physical activity guidelines for aerobic and muscle-strengthening exercise. The HP2030 program includes seven specific national health goals for physical activity in adults (30). With only 25% of adults and 20% of adolescents meeting the recommended weekly accumulation of health-related physical activity (30), Texas community colleges can have immediate and long-term impact by helping students learn and practice the knowledge, skills, and abilities to improve and maintain health through proper amounts of health-related physical activity. Physical activity can help prevent disease, disability, injury, and premature death (23). By being more physically active and meeting the requirements for health benefits, individuals can increase their healthy years of life while at the same time reduce the economic, social, and employment burdens on themselves, their families, and society. When community college students enroll and participate in health-related physical activity courses, they enhance their physical literacy.

Much like the HWPFL courses, most Texas community colleges do not have a graduation requirement for HRPA courses but the current status of HRPA courses in Texas community colleges is robust. There is strong evidence of offering various courses where students can engage in health-promoting physical activity. Forty-seven (94%) of the colleges offer a minimum of one HRPA course with most offering more than one and a variety of health-related physical activities. Although only two (4%) require such a course to meet AA or AS degree graduation requirements, almost all offer HRPA courses that can be taken as elective credit. Community colleges in Texas are in a very good position to educate students either through required or elective courses to improve and/or reinforce physical literacy in students for their immediate and long-term health.

The results of this study show that Texas community colleges have courses in the curriculum that can contribute to both health literacy and physical literacy of students during the transition from adolescence into young adulthood and beyond. Including HWPFL and HRPA courses as requirements for graduation is an interesting concept and making these a requirement has been shown to have positive effects on health and well-being during and after college (12, 13, 31). Making a HWPFL or HRPA course a graduation requirement would bring health-related learning and activities on par with other required coursework as seen in the Texas Core Curriculum (5). The argument can be made that contributing to the health and wellbeing of community college students in helping them be prepared for the workforce and as members of society, is just as important as preparing them for employment and being successful citizens. When courses of these types are not required, as seen in most Texas community colleges, then the enrollment of students in such elective courses should be highly prioritized and encouraged to contribute to individual health and well-being and empower students to take some responsibility for the influences on their personal health. This outcome would help to keep families, the community, and the workforce healthy and productive while reducing the economic burden that disease places on these same populations.

## Data availability statement

The original contributions presented in the study are included in the article/Supplementary material, further inquiries can be directed to the corresponding author.

## Author contributions

The author confirms being the sole contributor of this work and has approved it for publication.

## Conflict of interest

The author declares that the research was conducted in the absence of any commercial or financial relationships that could be construed as a potential conflict of interest.

## Publisher’s note

All claims expressed in this article are solely those of the authors and do not necessarily represent those of their affiliated organizations, or those of the publisher, the editors and the reviewers. Any product that may be evaluated in this article, or claim that may be made by its manufacturer, is not guaranteed or endorsed by the publisher.
